# Challenges in MRI-Guided Breast Biopsy and Some Suggested Strategies: Case Based Review

**DOI:** 10.3390/diagnostics12081985

**Published:** 2022-08-16

**Authors:** Chotai Niketa, Kathleen Ann Pang, Joon Wei Lim

**Affiliations:** 1 Radlink Diagnostic Imaging Center, #08-08, 290 Orchard Road, Singapore 238851, Singapore; 2 Yong Loo Lin School of Medicine, National University of Singapore, Singapore 119228, Singapore

**Keywords:** MRI breast, challenges, strategies

## Abstract

With the increasing use of MRI in clinical practice, the need for MRI-guided intervention is also increasing. Indeterminate lesions identified on MRI without mammographic or sonographic correlates will need to be approached under MRI guidance. MRI-guided biopsy is a skill that can be acquired with proper training and guidance. These procedures have their own set of challenges and issues; some of them are specific to the patient habitus in this region. Adequate knowledge and understanding of the challenges can help the radiologist to be better equipped to face these issues and solve them promptly during the procedure, thus increasing the overall success rate of the procedure. Not much local data from Asian countries is available on this front. This paper aims to share common challenges one may face while performing MRI-guided biopsy and share some tips and tricks to address these problems. Hopefully, this will help the readers achieve a higher success rate for MRI-guided interventions in their clinical practice.

## 1. Introduction

With the increasing usage of MRI in the clinical setting, there is a greater need for MRI-guided intervention as well [1,2]. The lesions detected on MRI with concerning features may need histological correlation [3]. If no definite correlate is identified on mammogram or ultrasound, then a biopsy needs to be performed under MRI guidance for such MRI-only lesions [2].

Several studies describe the feasibility and outcome of MRI-guided breast interventions, but most of them are from American or European countries. There is limited data available on MRI-guided breast biopsies in Asian countries. In this part of the world, there is limited use of MRI-guided intervention [4] due to its extremely high cost and limited availability [5] of this procedure and expertise.

An MRI-guided breast biopsy is advantageous as it allows biopsy of non-palpable breast lesions that are not detected on conventional imaging, including mammograms and ultrasound [6,7]. However, the procedure has its own unique set of challenges and limitations. Appropriate knowledge and understanding of these issues can enhance the overall success rate of the procedure [8] and boost the confidence of the radiologist performing it. In this article, we discuss various challenges that one may come across while performing MRI-guided biopsies and share some practical strategies to manage these challenges from our experience. Hopefully, this can help readers to pre-empt, and better equip themselves to solve difficulties they may face during the procedure. This could in turn improve the success rate of MRI-guided breast biopsies.

Of note, one must remember the different appearances of the breast [9] in its compressed versus non-compressed state and make use of adjacent landmarks to localize the correct target, especially if the lesion is small, or in the case of high background enhancement. It is advisable to check for adequate inclusion of the target within the compressed breast as much as possible before injecting the contrast and adjust the position, if needed, before the contrast injection.

Moreover, it is important to remind readers that though the medially located lesions may be approached from the medial side, it may be technically challenging. The longer lateral route may be opted for if the medial approach is too difficult.

The challenges discussed in-depth in this paper may be grossly divided into patient factors, lesion factors, technical factors, and others. Let us look into these challenges separately, along with case examples and suggested strategies.

### 1.1. Patient Factors

**Figure 1:**
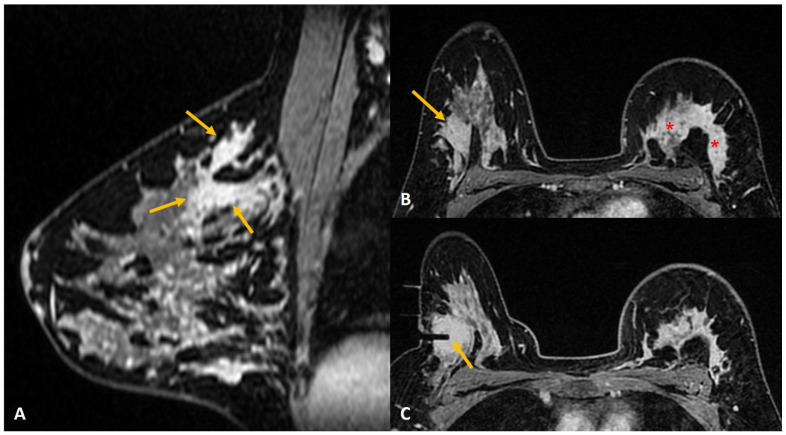
A 36-year lactating mother diagnosed with left breast inflammatory breast cancer underwent staging MRI. (**A**). Sagittal post contrast T1w fat-saturated image showed a segmental non-mass enhancement (yellow arrows) in the upper outer quadrant of the right breast. This was also distinctly FDG avid on PET scan (not shown here). (**B**). Axial staging scan showed diffusely enhancing mass in the left breast correlating with known cancer (red asterisks). Segmental non-mass enhancement was also seen in upper outer quadrant of the right breast (yellow arrow). (**C**). MRI-guided biopsy shows the tip of the obturator in correct location with surrounding post-biopsy hematoma (yellow arrow). Histology was reported as fibrocystic change.

(a) Anxious patient: Anxiety could come with the patient’s anticipation of pain or discomfort during core biopsy [10]. Claustrophobia, excessive movement, or other anxiety reactions in the MRI machine can affect the quality of images produced [11] and hence accurate targeting. Answering the patient’s queries and providing detailed explanations of the procedure, available alternatives, and possible complications can reduce the patient’s anxiety to some degree as the patient knows what to expect during the procedure [12]. Reassurance, comfort, and a good rapport can improve the patient’s cooperation during the procedure, which is pivotal for success. When needed, anxiolytics [13] or even sedation can be administered. Sedation can increase patient comfort, reduce motion artifacts, and decrease failure rates of the procedure due to premature termination [14]. At our practice, nearly 10% of patients opt for intravenous sedation under the care of an anesthetist. MRI-compatible monitors and a few other accessories may be needed for patient care. Minimizing patient anxiety not only improves patient satisfaction and comfort, but also the clinic’s efficiency and diagnostic capabilities [15].

(b) Lactating mothers (Figure 1): Although MRI carries no radiation risk, contrast-enhanced MRI is generally not recommended as the initial choice of investigation in lactating women. Only 0.04% of the intravenous dose of gadolinium-based contrast medium is being excreted in breast milk, of which less than one percent of the contrast medium excreted is being absorbed by the infant’s gastrointestinal tract [16]. As such, a decision to proceed with the MRI-guided breast biopsy may be taken if the benefit seems to be significantly higher. However, if the woman is concerned, the ACR suggests using a breast pump to express adequate breast milk before contrast-based procedures. After the procedure, she can be advised to stop breastfeeding and to express and discard the breast milk up to 24 h post-procedure [16].

In a lactating woman, the background parenchymal enhancement may significantly obscure small lesions. Also, intervention may be associated with a higher risk of infection and the risk of developing a milk fistula. A milk fistula is a rare potential complication of core or excision biopsy in a lactating woman [17]. Despite these risks, one may proceed with an MRI-guided biopsy when indicated with the utmost care to avoid infection, as a prompt diagnosis is important when suspecting breast cancer in a lactating mother [18]. The authors suggest optimum sampling in these women to reduce the risk of milk fistula. Tight compression after the procedure to close the tract can also play an important part.

**Figure 2:**
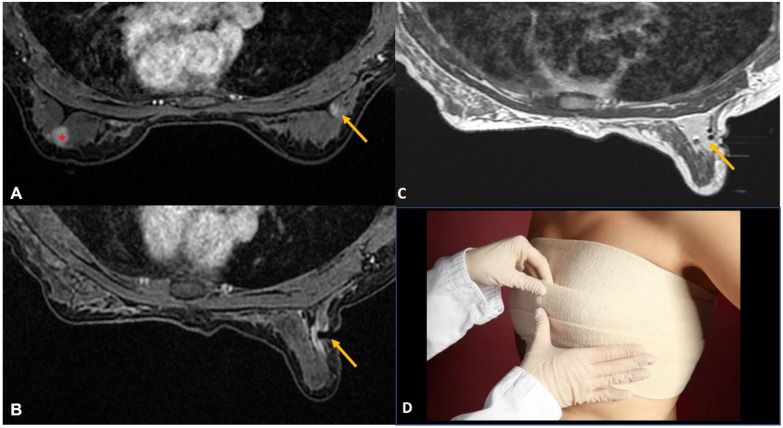
(**A**). Staging MRI in a woman with known left breast cancer (red asterisk) showed focal non-mass enhancement in the right breast (yellow arrow). (**B**). MRI-guided biopsy to rule out contralateral synchronous cancer showed the tip of the obturator at the right location (yellow arrow). (**C**). Post-biopsy non-fat saturated image showed a large hematoma at the biopsy site almost occupying the upper outer quadrant (yellow arrow). (**D**). Tight compression bandage applied to the breast after the biopsy reduces the risk of further bleeding significantly.

(c) Hematoma formation (Figure 2): MRI-guided biopsy, being a vacuum-assisted biopsy procedure needing a large bore needle size (generally 8-10G), may cause some bleeding and hematoma formation at the site of biopsy. An inquiry should be made in every patient for any known bleeding disorders or history of anticoagulant therapy. In routine cases, the hematoma can be managed with adequate post-procedure local compression. Additional tight breast wrap at the biopsy site can be provided to the patient for continued compression for the next 24–48 h. Aspirating a large hematoma with the use of a vacuum before deploying the marker clip may be helpful in some cases. In the case of an arterial injury leading to significant bleeding, injection of thrombin in the biopsy cavity may help to control active bleeding.

Although some papers suggest that no significant complications were experienced in patients on anticoagulation therapy post-MRI-guided biopsies [19,20], there may still be a risk of hematoma formation due to the large sample size and, hence, whenever possible, the anti-coagulant therapy should be withheld during the procedure time. On the other hand, it is important to understand that, for any reason, if anticoagulant therapy cannot be stopped in a patient needing an MRI-guided biopsy, one can proceed with the biopsy with added precautionary measures. This is more so in a patient with an INR within the therapeutic range within two weeks of the procedure [21]. Therefore, checking the patient’s International Normalized Ratio (INR) prior to the biopsy day may be helpful. Depending on the INR, discussions with clinicians can be made to consider continuing, withholding, or bridging blood thinners if the patient cannot be off anticoagulant therapy [22]. When possible, stopping anticoagulant medicine for at least 5–7 days before the biopsy reduces the bleeding risk. Overall, these contraindications should be evaluated with the radiologist before the scheduled MRI biopsy day, and one must follow institutional guidelines.

**Figure 3:**
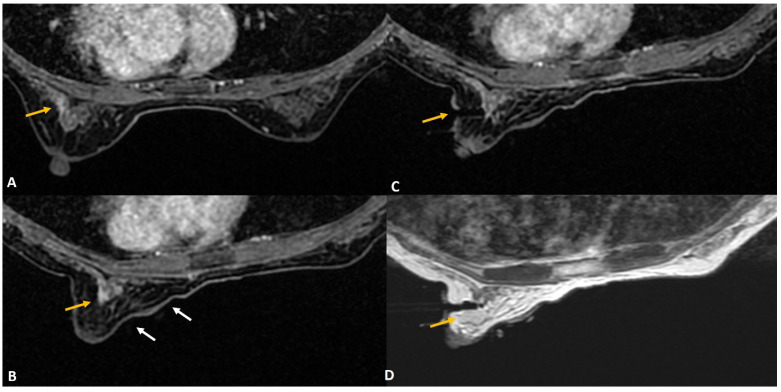
(**A**). MRI was performed in a woman with architectural distortion on mammogram detected during screening without sonographic correlate. A small enhancing correlate was identified on MRI (yellow arrow). (**B**). The breast size was extremely small. After removing the padding from the breast coil, the patient was tilted to obtain the target site in the grid hole. Additional padding was applied on the medial aspect (white arrows) to obtain optimal breast thickness making the procedure feasible and the target lesion (yellow arrow) accessible. (**C**). Tip of the obturator was noted at the target site (yellow arrow) and biopsy was performed using blunt tip Petit needle. (**D**). Post-biopsy hematoma (yellow arrow) is noted with adequate sampling. Histology was reported as radial scar.

(d) Small Breasts (Figure 3): This is a common issue in this part of the world, with a cup size of A or AA encountered in nearly a third of patients. Small breasts pose a challenge when the mid to posterior part of the breast does not fall into the grid. Adequate care taken while positioning the patient can contribute significantly to procedural success. Removing the chest pad from the coil allows more posterior breast tissue to fall into the grid. In addition, tilting the patient into an oblique position allows lesions in the posterior breast to fall into a more accessible location for biopsy. Using a Petit needle or half-aperture size needle with extra cores can be used in a thin breast with borderline thickness to avoid injury to the skin or chest wall.

### 1.2. Lesion Factors

Characteristics of the lesion such as its size, location, and mobility can make the procedure challenging.

**Figure 4:**
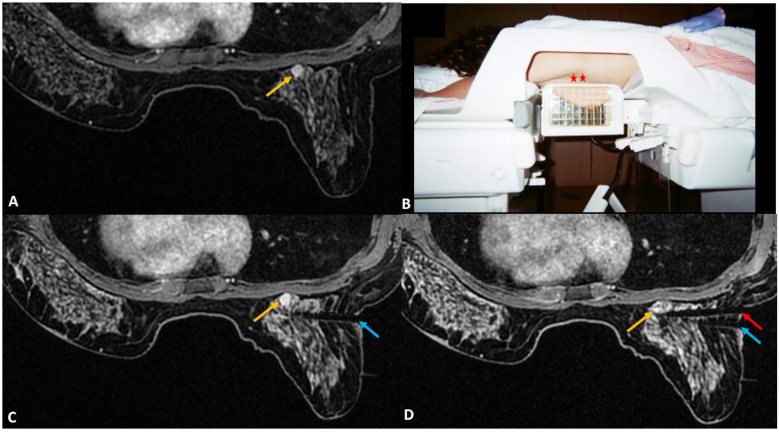
(**A**). In a woman with newly diagnosed left breast cancer, staging MRI identified a 6 mm indeterminate enhancing mass (yellow arrow) in the right breast located posteriorly close to chest wall. (**B**). During biopsy small dead space (red asterisks) is always noted close to the chest wall that lies beyond the grid. (**C**). For biopsy, the introducer was inserted through the posterior most hole in the grid (blue arrow) close to the lesion (yellow arrow). The needle failed to reach the target adequately. A second introducer guide was inserted outside the grid (in the area of red asterisks), close to the chest wall using free-hand direct puncture. (**D**). The posterior needle (red arrow) was seen to adequately reach the target and biopsy was completed successfully. The histology was reported as lobular neoplasia.

**Figure 5:**
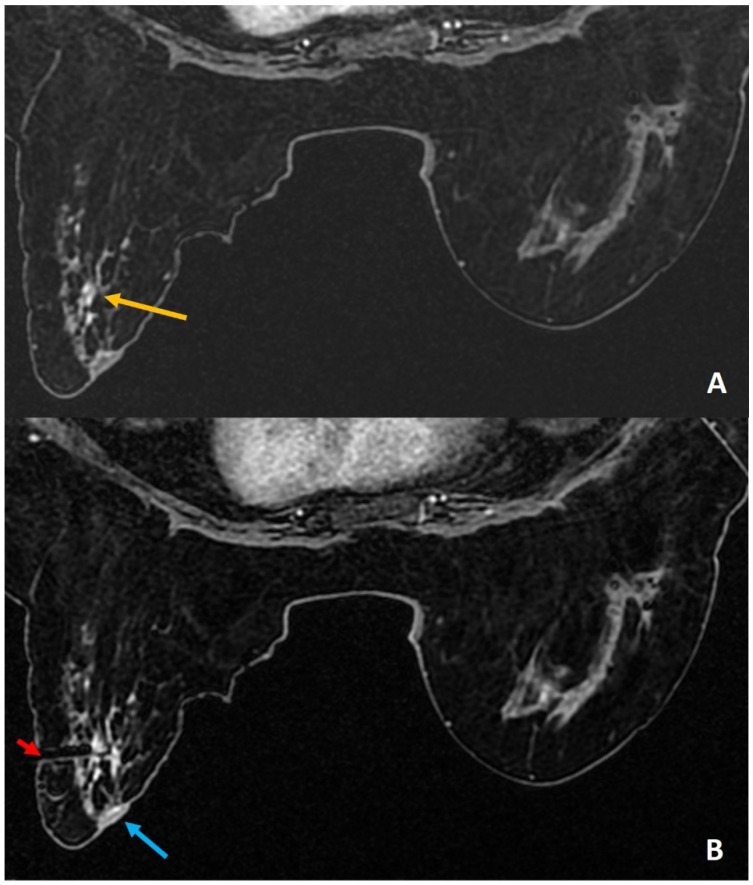
(**A**). High-risk screening MRI reported clumped linear non-mass enhancement (yellow arrow) in the left retroareolar region. (**B**). During biopsy, rolling of the breast with nipple directed away (blue arrow) from the skin entry (red arrow) could help to avoid injury to the areola. The histology was reported as atypical ductal hyperplasia.

**Figure 6:**
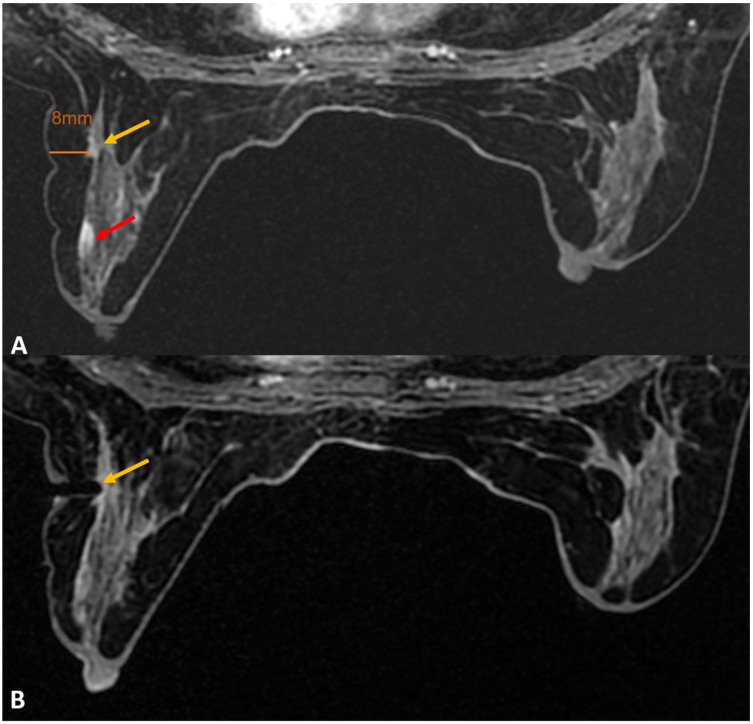
(**A**). Known right breast DCIS in anterior third depth (red arrow). Small indeterminate enhancing mass in the posterior breast (yellow arrow) was for biopsy to assess the extent of DCIS to plan the surgery. The target was seen 8 mm from the skin, as shown. The tip of the obturator is seen at the target. A normal 20-mm aperture needle would leave part of the trough outside the skin and can lead to injury to skin and air leak during biopsy. (**B**). A small-aperture, blunt tip Petit needle (12 mm aperture) (yellow arrow) was used for safety and reducing the risk of skin laceration.

(a) Location of Lesion:

(ai) Posterior lesions (Figure 4): Lesions in the posterior breast that are close to the chest wall or abutting the pectoralis major can be difficult to access. This is even more so in smaller breasts as the target lesion may lie outside of the grid. In addition to the approaches to smaller breasts discussed above, we suggest a few more strategies that readers may find useful. Firstly, the breast lesion may sometimes fall out of the grid due to its extremely posterior location close to the chest wall. Advancing the needle into the breast posterior to the grid, along with adjusting the depth of the needle, can resolve this issue. One must remember that due to the absence of support by the grid in this location, the trocar may be unstable and extra care should be taken to obtain accurate sampling. Secondly, a freehand insertion of the needle can be performed with the breast lying freely in the coil without using the grid and compression. The needle can then be advanced and angled freely. Some compression can be applied to stabilize the breast during the process [22]. The pillar and post method allows wider accessibility of breast lesions.

(aii) Periareolar lesion (Figure 5): As the areola is more vascular in nature, a biopsy of the lesions in the peri/retroareolar region poses a significant bleeding risk. Piercing the areola is avoided as much as possible for reasons of cosmesis, reduction of pain, and reduction of bleeding. Rolling the breast such that the nipple-areola complex is away from the point of skin entry and the use of additional padding at the anterior aspect of the breast can increase the thickness of the anterior breast and thus aid access to the periareolar lesion for biopsy.

(aiii) Superficial lesions (Figure 6): A superficially located breast lesion may pose a challenge when using the regular needle size due to the increased risk of cutting the skin during the biopsy. This may lead to air leaks and loss of vacuum. Firstly, raising a good-sized skin wheal using a local anesthetic agent may allow more depth for the needle to be inserted so as to avoid unwanted trauma to the skin [22]. Additionally, using a smaller aperture needle may be considered. Another option could be to adjust the depth by a few millimeters beyond the target, allowing the lesion to fall at the periphery of the aperture.

**Figure 7:**
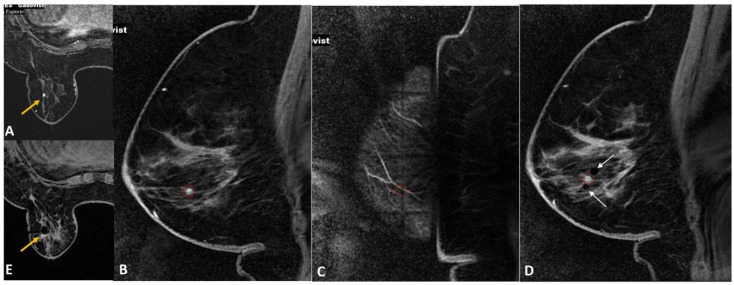
(**A**). High-risk screening in a BRCA 2 woman showed a new 4-mm enhancing focus in the left breast (yellow arrow). (**B**). Sagittal image on the biopsy day showed the target focus enhancing well (red circle). (**C**). During targeting, the entry point was noted to fall on the “cross” of the grid (red circle), which blocked the ideal entry site. (**D**). In this case, we performed dual-needle targeting though diagonally adjacent holes. Two obturators in these holes (white arrows) are seen adjacent to the target (red circle). Selected directed sampling through both these needles towards the target would increase the chance of getting the tiny lesion biopsied adequately. (**E**). Post biopsy hematoma cavity (yellow arrow) was seen at the target site with non-visualization of the target suggesting adequate sampling. The histology was proven as 3-mm invasive tubular carcinoma.

**Figure 8:**
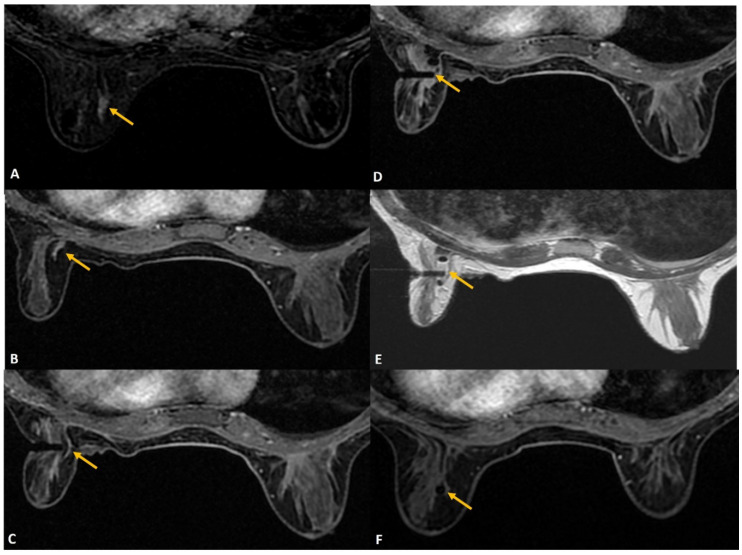
(**A**). Screening MRI in a high-risk patient showed a new indeterminate focal non-mass enhancement (yellow arrow) in left breast. (**B**). During the biopsy, the enhancing lesion (yellow arrow) was seen compressed and pushed along posterior aspect. (**C**). The lesion (yellow arrow) was displaced medially by the obturator of the biopsy guide. (**D**). The needle was pushed beyond the target and then withdrawn to correct depth (yellow arrow). This strategy helped to place the needle well in relation to the target lesion. (**E**). Post-biopsy hematoma (yellow arrow) is noted. (**F**). Follow up MRI after a year confirmed appropriate location of the marker clip with lack of enhancement in this region (yellow arrow). The histology of this lesion was reported as sclerosing adenosis.

(b) Small-sized lesions obscured by grid (Figure 7): Occasionally, a small lesion to be biopsied may fall on a “cross” of the grid during the biopsy. In this case, the grid obscures the ideal entry site and offsets the needle. Hence, using two diagonally placed entry sites from the adjacent holes, along with directed sampling, may further enhance the chance of success. Targeted sampling with extra cores directed towards the lesion may help in this situation.

(c) Lesion Movement (Figure 8): Lesion movement may be due to patient movement, inadequate breast compression, or skin indentation. Firstly, the patient must be comfortable and well-informed of the procedure to avoid startling her. If needed, readjust the patient’s position. Adequate breast compression is needed to immobilize the breast well, and a good skin incision may avoid excessive skin indentation. Occasionally, inserting the needle beyond the target and then withdrawing it to the correct depth may help to get a perfect location of the needle aperture at the targeted site.

### 1.3. Technical Factors

**Figure 9:**
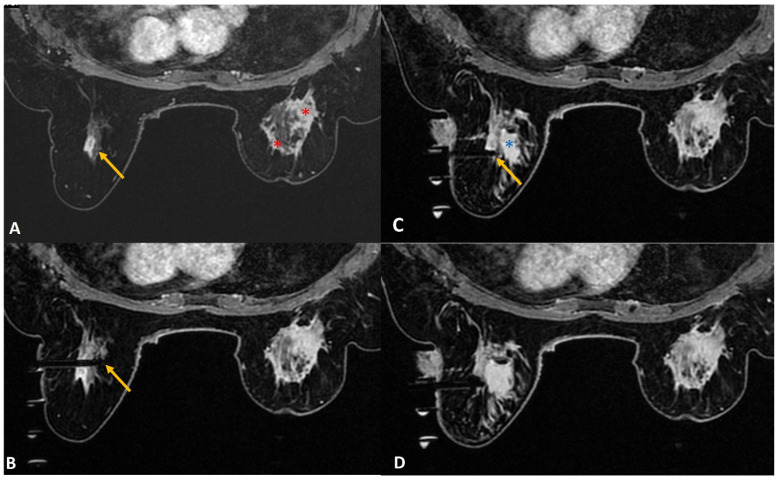
(**A**). In a patient with recently diagnosed right breast cancer (red asterisks), staging MRI showed a 9-mm enhancing mass (yellow arrow) in the left breast at 12 o’clock. (**B**). During biopsy, the tip of the obturator was seen beyond the target (yellow arrow). This was because the wrong needle size was mistakenly selected on CAD software producing wrong ‘z’ axis/depth. (**C**). The obturator depth was then adjusted to correct depth with its tip at the lesion (yellow arrow). By this time, a significant size of hematoma (blue asterisk) was noted in the inner half of the breast from arterial injury. (**D**). The biopsy of the target was completed successfully and the histology from the left breast lesion was reported as high-grade DCIS with comedonecrosis.

(a) Non-Enhancement of Lesion: To minimize the incidence of lesion non-enhancement, radiologists can check if contrast was indeed injected. If injected, one must rule out extravasation of contrast into the soft tissues at the injection site. If adequate enhancement of the heart is confirmed, then measures such as taking delayed sequences and reducing the breast compression, allowing greater flow in the breast, may be attempted. If the lesion shows minimal or faint enhancement, then making use of landmarks such as vessels, biopsy clips, or fat planes may be considered to ensure correct targeting. If all these considerations fail to enhance the lesion, it is recommended to withhold the biopsy and request a six-month follow-up MRI [23]. A cancellation rate of nearly 8–13% is reported in the literature for MRI-guided breast biopsies [24,25]. Though it is likely that the majority of these lesions may represent fluctuating physiologic background enhancement, non-enhancement on the day of biopsy does not guarantee benignity. It has been shown that there is approximately a 3.5% chance of cancer in these patients on follow-up [26]. Hence, as per the ACR recommendations, we must ensure that the patient returns for close follow-up in 3–6 months. If the lesion reappears on follow-up MRI, or if the lesion is seen larger or with suspicious features, then the biopsy should be rearranged.

(b) Improper targeting: The risk of improper targeting is higher with manual methods compared to using commercially available software. Incorrect localization of the target may be due to the selection of the wrong laterality (left versus right localization chart) while calculating the coordinates, or from the improper translation of calculated x and y axes coordinates from the “image” view to the “patient” view. An improper z-axis (depth of target) can result from entering the wrong needle size on computer-aided detection (CAD) software (Figure 9).

**Figure 10:**
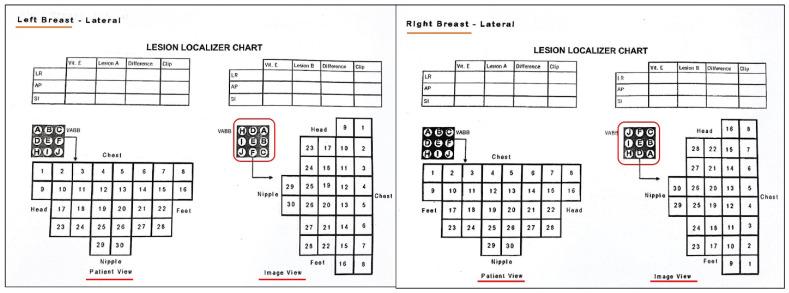
Examples of the charts used for manual targeting during MRI breast biopsy. Please note that location of various co-ordinates differs with the laterality and approach used for the case. Translation of the target from image view to patient view is also a critical step and a mistake in this may lead to wrong targeting.

Careful use of correct laterality and approach, as well as accurate translation of calculated target from the “image” view to the “patient” view, are of utmost importance, while using the manual targeting method. Note that the patient views differ for the left and right breasts, as demonstrated in Figure 10.

**Figure 11:**
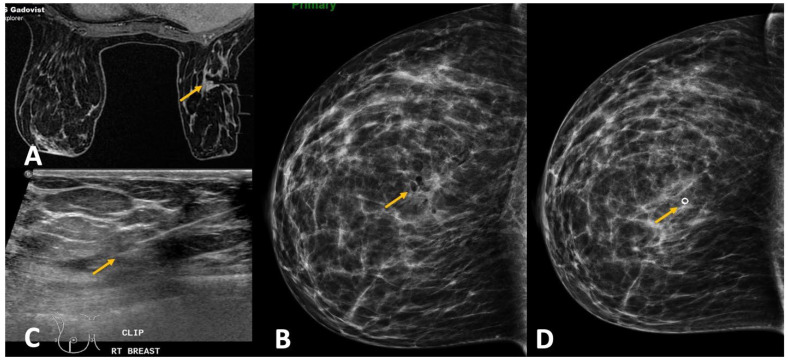
(**A**). MRI-guided biopsy of right breast for a 7 mm suspicious mass (yellow arrow) was performed to rule out synchronous cancer. The procedure was uneventful. (**B**). Post-biopsy check mammogram showed absent marker clip and air within the biopsy cavity (yellow arrow). An expedited pathology report was requested and returned as invasive ductal carcinoma. (**C**). Ultrasound revealed a small hematoma at the biopsy site and hence an ultrasound guided marker clip was deployed (yellow arrow). (**D**). Repeat check mammogram confirmed the marker clip at the expected location (yellow arrow).

(c) Non-Deployment of Clip (Figure 11): A rather uncommon challenge is the non-deployment of the clip. This may be due to technical errors, such as using an improper device or the wrong technique. Occasionally, the clip may remain adherent to the tip of the sheath. Upon withdrawing the sheath at the end of the procedure, the adherent clip may then be pulled out together with the sheath. Aspiration of a large hematoma post-biopsy may also be associated with extrusion of the clip. Other times, a large hematoma formed may lead to the displacement of the clip by preventing adherence of the clip to tissue or by mass effect [27]. To reduce the risk of a large hematoma formation, which can result in clip migration, a few techniques can be used. Firstly, lavaging the biopsy cavity adequately after the sampling before deploying the clip. Secondly, aspirating a large hematoma through the sheath prior to deployment of the clip. Thirdly, the use of a vasoconstriction agent along with lidocaine during biopsy may reduce the bleeding risk. A check mammogram is generally performed after the biopsy procedure to document adequate clip deployment, in addition to post-biopsy hematoma. If it fails to show the clip, this confirms the failure of the deployment. An alternative modality using ultrasound-guided deployment of a new clip may be extremely useful to annotate the site of biopsy [28].

### 1.4. Other Challenges

**Figure 12:**
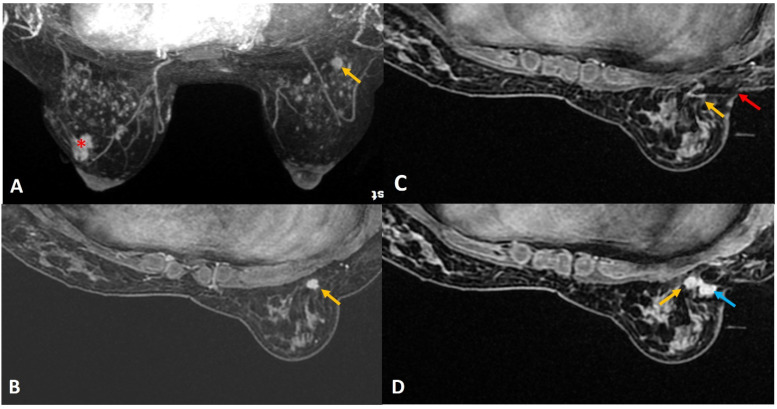
(**A**). MIP image from staging scan of a woman with diagnosed left breast cancer (red asterisk) showed indeterminate lesion in the right breast (yellow arrow). (**B**). In absence of definite sonographic correlate, MRI-guided biopsy was arranged. The target was noted outside the grid compression (yellow arrow). (**C**). A free-hand direct insertion of the guide shows the tip of the obturator (red arrow) exactly within the target lesion (yellow arrow). The histology returned as normal breast tissue that was considered discordant. (**D**). Retrospective review of the post biopsy image showed the target lesion (yellow arrow) along the medial aspect of the hematoma (blue arrow). Possibility of accidental partial withdrawal of the needle during the vacuum procedure was considered, thus missing the target completely. This was communicated with the surgeon and excision biopsy was recommended in view of radiology–pathology discordance. Final surgical histology was reported as 7-mm invasive ductal carcinoma.

(a) Radiology-pathology discordance (Figure 12): After a successful biopsy, it is equally essential to review the histology [29] and issue a radiology-pathology correlation report. This allows discordant results to be picked up without delay. When there is discordance, it is advisable to communicate with the surgeon to suggest the next step in the management of the patient. A possible next step may be a repeat biopsy or an excisional biopsy.

## 2. Conclusions

In conclusion, the use of MRI-guided intervention has been increasing. The success of MRI-guided procedures depends on multiple factors. One factor is teamwork among the surgeon, radiologist, staff nurse [23,30,31,32], and MRI technician. Another factor is the radiologist’s familiarity with the steps of the procedure and pre-procedural planning [33] to approach the target in the given patient. A clear, calm mind while performing the procedure can reduce the challenges significantly. Conscious, deliberate efforts in the preparation and positioning phases will improve the efficacy of the procedure. The concepts of directional sampling, the importance of sampling adequacy, and radiologic-pathologic correlation should also be equally emphasized. Some of the most common challenges that one may face during the procedure have been enumerated here along with strategies to handle them. We wish to inspire our readers to apply our proposed strategies in their clinical practice when faced with MRI-guided breast biopsy challenges. Hopefully, this achieves higher success rates, which translates to an overall improvement in patient outcomes.
